# Editorial: Third-Generation Neuroimaging: Translating Research into Clinical Utility

**DOI:** 10.3389/fpsyt.2016.00170

**Published:** 2016-10-12

**Authors:** André Schmidt, Stefan Borgwardt

**Affiliations:** ^1^Department of Psychiatry (UPK), University of Basel, Basel, Switzerland; ^2^Department of Psychosis Studies, Institute of Psychiatry, Psychology and Neuroscience, King’s College London, London, UK

**Keywords:** psychiatry, neuroimaging, prediction, transition, remission, treatment responses

**The Editorial on the Research Topic**

**Third-Generation Neuroimaging: Translating Research into Clinical Utility**

As yet, no reliable structural or functional brain marker has been univocally associated with any psychiatric disorder, and no clinical applications have been developed in psychiatric neuroimaging (1–4). There is thus urgent need of psychiatric imaging to move toward third-generation paradigms. First-generation psychiatric neuroimaging focused on simple structural brain alterations associated with the neurobiology of the illness. These early studies adopted imaging methods mainly including computerized tomography (CT) to investigate brain size (5). Second-generation psychiatric neuroimaging studies benefited from more sophisticated techniques, which included structural techniques such as magnetic resonance imaging (MRI) and diffusion tensor imaging (DTI), functional approaches such as task-related or resting-state functional magnetic resonance imaging (fMRI), and electroencephalography (EEG) and neurochemical measurements like positron emission tomography (PET), magnetic resonance spectroscopy (MRS), and single-photon emission computed tomography (SPECT). However, by using these powerful non-invasive measurements, psychiatric imaging needs to move away from simple investigations of the neurobiology underling the early phases of psychiatric diseases in order to translate imaging findings into daily clinical routines, targeting clinical outcomes including transition, remission, and response to preventative treatment scenarios (1, 2, 6–11).

The aim of this research topic is to provide the field with an overview of current third-generation neuroimaging approaches in translational psychiatry that is hoped to improve and create therapeutic options for psychiatric diseases. This Research Topic includes articles indicating the potential of specific network connectivity analyses for inferring on the pathophysiological mechanisms of schizophrenia (Silverstein et al.), autism spectrum disorder (Crippa et al.), or suicidal behavior (Serafini et al.), or how they may help to predict the cognitive enhancing effect of pharmacological agents across disorders (van Amelsvoort and Hernaus) or psychotherapeutic interventions in patients with ADHD (Bachmann et al.) and schizophrenia and comorbid substance misuse problems (Wojtalik et al.). However, one article also emphasizes the importance of further second-generation imaging to investigate specific symptoms in a systematic manner before third-generation imaging can be informed (de Cates and Broome). Further contributions are suggesting advanced optical topography (Ho et al.), ^18^F-FDG PET (Kowoll et al.), or EEG microstates (Rieger et al.) or beta oscillation analyses (Ghorashi and Spencer) as promising approaches to guide third-generation imaging across disorders (Ho et al.) or in schizophrenia [Ghorashi and Spencer; Rieger et al.], while others argue for the fusion of multimodal imaging modalities (Bellani et al.; Chiapponi et al.; O’Halloran et al.). Multimodal approaches, which integrate brain activation and connectivity patterns with metabolic measurements, are also proposed to gain a better understanding of the neuropathology underlying basic symptom in psychosis (Schultze-Lutter et al.). The current Research Topic also reveals the clinical utility of machine learning methods using multimodal imaging data in identifying individuals at high risk for psychosis (Valli et al.) and predicting outcomes across psychiatric populations (O’Halloran et al.; Schnack and Kahn), as well as of real-time fMRI (Dyck et al.; Fovet et al.; Gerin et al.) in treating symptoms of PTSD (Gerin et al.) and auditory–verbal hallucinations in schizophrenia (Dyck et al.; Fovet et al.). Finally, this topic outlines a theoretical framework how Hierarchical Bayesian Models of functional neuroimaging data may help to establish diagnostic test in autism spectrum disorder (Haker et al.).

This issue is intended to provide a useful framework for further third-generation imaging investigations aiming at predicting clinical outcomes, such as transition, remission, and treatment responses in early phases of different psychiatric diseases. These types of analyses might help to improve and develop novel therapeutic scenarios. We would like to thank all the authors and reviewers for their valuable contributions, as well as the Editorial Office for their help in the editing process.

## Author Contributions

All authors listed have made substantial, direct, and intellectual contribution to the work and approved it for publication.

## Conflict of Interest Statement

The authors declare that the research was conducted in the absence of any commercial or financial relationships that could be construed as a potential conflict of interest.
